# A family-based multimedia intervention to enhance the uptake of colorectal cancer screening among older South Asian adults in Hong Kong: a study protocol for a cluster randomized controlled trial

**DOI:** 10.1186/s12889-019-6995-7

**Published:** 2019-05-28

**Authors:** Winnie K. W. So, Bernard M. H. Law, Kai Chow Choi, Dorothy N. S. Chan, Carmen W. H. Chan

**Affiliations:** The Nethersole School of Nursing, The Chinese University of Hong Kong, Shatin, Hong Kong, China

**Keywords:** Colorectal cancer, Cancer screening, South Asians, Multimedia intervention, Family-based

## Abstract

**Background:**

Colorectal cancer (CRC) screening, such as fecal occult blood test (FOBT), is an effective way to prevent CRC, one of the most common cancers worldwide. However, studies found that South Asian ethnic minorities tend not to utilize CRC screening, whose importance on CRC prevention shall be educated among those from ethnic minorities, especially older adults. The purpose of this study is to develop and implement a family-based, multimedia intervention to augment the knowledge of CRC prevention among older South Asian adults in Hong Kong and enhance their motivation for undergoing FOBT. The acceptability and effectiveness of the intervention will be assessed using the Reach, Effectiveness, Adoption, Implementation, Maintenance (RE-AIM) framework.

**Methods:**

A cluster randomized controlled trial will be carried out. Three hundred and twenty South Asian dyads, comprising an older adult aged between 50 and 75 and a younger family member aged between 18 and 49, will be recruited in ten districts in Hong Kong through community organizations that provide support services for South Asians in local communities. Dyads will be randomly allocated to either the intervention or wait-list control group. Intervention dyads will receive intervention, whose contents are based on the health belief model, via multiple forms of media including PowerPoint presentation, video clip and health information booklet. Control dyads will receive intervention after post-intervention data are collected. For dyads in both groups, an appointment with a family doctor will be arranged for those willing to undergo FOBT. Outcomes will be assessed at baseline and post-intervention. Data will be analysed using the Generalised Linear Models Procedure in an intention-to-treat manner.

**Discussion:**

Findings of this study will provide evidence of the benefits of utilizing multimedia and family-based approaches in intervention development to enhance the effectiveness of health promotion interventions for ethnic minorities. Further, the findings would provide reference to the potential incorporation of the intervention in the existing support services for South Asian ethnic minorities in local communities.

**Trial registration:**

This trial is registered at the ISRCTN Registry (ISRCTN72829325) on 19th July 2018.

**Electronic supplementary material:**

The online version of this article (10.1186/s12889-019-6995-7) contains supplementary material, which is available to authorized users.

## Background

Colorectal cancer (CRC) is currently one of the most prevalent cancers, with more than 1.36 million new cases reported in 2012 worldwide [1]. Notably, CRC prevalence rate has been generally increasing over the past decades, with the projection of incidence at over 2.2 million by 2030 [2]. In Hong Kong, CRC appears currently as the most common cancer in terms of incidence, with more than 5000 new cases reported in 2015 [3]. More importantly, the risk of CRC rises with age, with the majority of CRC onset at the age of 50 or above [4]. The implementation of effective preventative measures against CRC development, especially among older adults, is of utmost importance to help control the growing prevalence of CRC worldwide. CRC screening strategies, such as colonoscopy and fecal occult blood test (FOBT), are proved to be effective in CRC prevention and reduction of CRC mortality [5, 6], primarily by enabling early diagnosis, thereby increasing the chance of cure through effective cancer treatment. In the light of the benefits of CRC screening in checking against CRC development, CRC screening programs have been developed and implemented worldwide [7]. Likewise, the Hong Kong government has also launched a pilot scheme to encourage utilization of CRC screening services among individuals aged between 50 and 75, who are encouraged to visit their family doctors for an FOBT. Those with detectable amount of blood in their stool will be asked to undergo colonoscopy examination for further assessment of signs of CRC [8].

Despite the availability of CRC screening programs to the public, previous studies showed that ethnic minorities tend not to undergo CRC screening, as evidenced by the lower utilization rate of CRC screening services by ethnic minorities compared to the general population [9–15]. Indeed, a recent systematic review also revealed that being a member of the ethnic minorities is one of the barriers that discouraged the utilization of CRC screening [16]. Previously, studies had revealed a number of factors that could contribute to this phenomenon, including lack of knowledge of CRC and its preventive measures, embarrassment, lack of recommendations on physicians and limited access to healthcare [17–20]. These findings suggest that development and implementation of educational interventions dedicated to enhancing the knowledge of ethnic minorities on CRC prevention, as well as their self-efficacy and motivation to utilize CRC screening would be necessary to increase their uptake of screening. With South Asians being one of the largest and fast-growing populations in Hong Kong [21], they should be targeted in such health-related interventions to address the ethnic disparities in CRC screening utilization in Hong Kong [14].

Previously, studies were conducted in various Western countries to evaluate the effectiveness of certain interventions in reducing the existing disparity in the utilization of CRC screening services between ethnic minorities and the general population. A systematic review published by Naylor et al. had summarized their findings [22]. Overall, the review demonstrated that interventions involving the provision of education on CRC and its preventive measures to ethnic minorities via culturally- tailored materials, alongside navigation services, would enhance the uptake of CRC screening or adherence to CRC screening guidelines among these individuals. Likewise, systematic reviews on CRC screening interventions developed specifically for Latino [23] and Hispanic [24] ethnic minorities yielded similar findings, showing that the provision of education and navigation are key to a higher uptake of CRC screening. Notably, they also highlighted the importance of using culturally-tailored educational materials in enhancing the effectiveness of interventions. Indeed, the role of culturally-tailored interventions in enhancing the knowledge of CRC and CRC screening uptake among ethnic minorities was also demonstrated in a recent study with Hispanics in rural communities in the United States [25]. The inclusion of the above features should therefore be considered in the development of future interventions for improving CRC screening uptake among ethnic minorities.

Nevertheless, to further improve the efficacy of such interventions targeting older adults, strategies such as the involvement of family members in intervention should also be considered. A previous review had demonstrated the benefits of family involvement in enhancing the effectiveness of health-related interventions dedicated to improving health outcomes of older adults with diabetes [26]. More importantly, family support was shown in a qualitative study to be a major factor that may modify the CRC screening behaviours of older South Asian adults [27]. For example, older adults were more reliant on their younger family members in interpreting written materials for health information, and the participation of older adults in CRC screening programs was largely dependent on the recommendations by their descendants. The education of younger family members on the importance of screening in CRC prevention could increase the likelihood for them to recommend screening to their parents, thereby enhancing the screening uptakes of older adults. Therefore, the involvement and participation of younger family members may be conducive to interventions centring older adults.

Despite the availability of studies evaluating the effectiveness of interventions on enhancing the uptake of CRC screening among ethnic minorities, there is currently a scarcity of such studies on South Asians. As cultural relevance of intervention materials appears an important factor in the efficacy of health-related interventions for ethnic minorities, intervention materials used in previous studies on ethnic groups may not necessarily be applicable to the intervention on South Asians. There is a research gap in intervention development that addresses ethnic disparities in CRC screening uptake within communities. To address this gap, we propose to develop a family-based multimedia intervention for older South Asian adults in Hong Kong that educate them and their younger family members on CRC and the importance of screening in disease prevention. The intervention, carried out through partnership with various local South Asian community organizations, aims to enhance participants’ knowledge of CRC and increase their self-efficacy and motivation for undergoing FOBT via a local family doctor. Findings of this study will inform healthcare providers, both in Hong Kong and worldwide, of potential utility of this intervention in enhancing CRC screening uptake among older South Asian ethnic minorities for effective CRC prevention, which could potentially help reduce the global prevalence of the disease.

### Objectives

The objectives of this study are as follows:

- To develop and implement a family-based, culturally sensitive and linguistically appropriate multimedia intervention dedicated to enhancing the self-efficacy and motivation of older South Asian adults in Hong Kong to undergo FOBT.
- To evaluate whether this intervention is effective in encouraging the uptake of CRC screening and FOBT among older South Asian adults in Hong Kong through the five dimensions in the Reach, Effectiveness, Adoption, Implementation, Maintenance (RE-AIM) framework developed by Glasgow et al. in 1999 [28]. Details of the parameters in each RE-AIM dimension are outlined in the ‘Outcome Measures’ section below.
- To assess whether this intervention is acceptable to South Asian families in Hong Kong.

## Methods

**Fig. 1:**
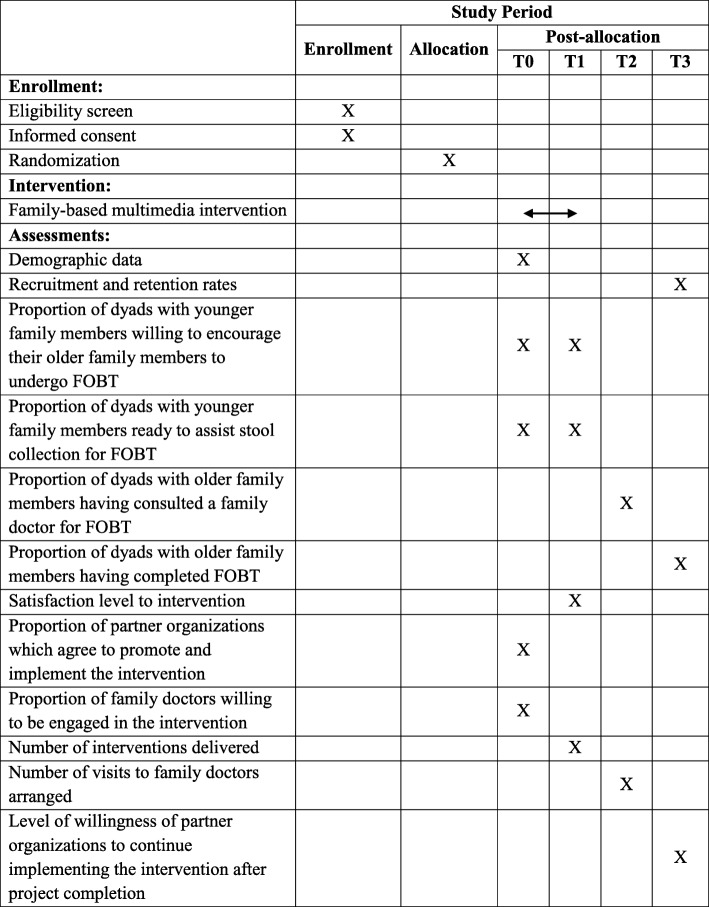
The SPIRIT Figure

The preparation of this study protocol follows the guidelines set out by the Standard Protocol Items: Recommendations for Interventional Trials (SPIRIT) checklist. The completed SPIRIT checklist is shown as Additional file 1. A SPIRIT figure is also provided as Fig. 1 to demonstrate the flow of the study.

### Design

This study will utilize a cluster randomized controlled trial design, which includes a wait-list control group.

### Study settings

Interventions will be conducted in various South Asian community centres, ethnic minority associations and non- governmental organizations that provide support services for South Asians within local communities in ten districts in Hong Kong. Partnership with these organizations will be sought prior to the implementation of the intervention, as described below. These organizations are referred as ‘partner organizations’ hereafter.

### Participants

A total of 320 dyads of older South Asian adults and their younger family members will be recruited for the study. The inclusion criteria for older adults include 1) having a South Asian origin (either Indian, Pakistani or Nepali), 2) aged between 50 and 75, 3) having a younger family member or relative aged between 18 and 49, and 4) residing in Hong Kong during subject recruitment. Those participated in our previous community-based project titled ‘A community health worker-led multimedia intervention to increase cervical cancer screening uptake among South Asian women: A randomized controlled trial’ will be excluded.

### Sample size estimation

Sample size calculation of this study is based on the findings of a previous study on the implementation of an intervention aiming to enhance CRC screening uptake among African Americans, under which the difference in CRC screening uptake between the intervention and control groups at six-month follow up was compared [29]. The authors observed a difference of 16.2% in the proportion of intervention and control participants who had undergone screening. To reject the null hypothesis that no difference in screening uptake exists between the two groups at a 5% level of significance, both groups require 126 dyads to achieve a power of 80%. To reach an attrition rate of 20%, a total of 320 dyads shall be recruited, with 160 dyads in each group.

### Subject recruitment

Recruitment of South Asian dyads will be carried out at partner organizations within local communities in Hong Kong. Prior to the implementation of intervention, our research staff will approach potential partner organizations to seek their support in subject recruitment. These organizations will be asked to distribute a subject recruitment notice amongst their South Asian members in form of posters and social media posts. Persons-in-charge of these organizations will also be invited to contact their members for taking part in the study. Permissions to recruit potential dyads during the social activities under partner organizations will also be sought. Potential dyads interested in joining the study will be screened for eligibility before enrolment.

### Randomization

A cluster randomization approach will be utilized in this study. In previous community-based projects, we had identified ten districts in Hong Kong populated with a large number of South Asians. These districts will be randomized into either the intervention or wait-list control group, with five districts allocated to each group respectively. Partner organizations located in each district will be allocated to either group based on the outcome of group allocation of respective districts. Sealed and numbered envelopes will be used during group allocation to ensure concealment. To ensure successful recruitment of 320 dyad participants for the study, we aim to recruit 50–60 dyad participants in each district.

Randomization will be conducted by a research staff member independent from the research team so that outcome assessors and study investigators will be blinded to group allocation. However, owing to the use of a wait-list control group in this study, it will not be possible to blind the dyad participants.

### Intervention

#### Development of intervention

The multimedia intervention developed in this study will involve dissemination of knowledge of CRC and the importance of CRC screening to South Asian dyad participants via a combination of intervention materials including a PowerPoint presentation, a video clip and a health information booklet. Prior to the development of these materials, an advisory panel will be formed, comprising health professionals, a community centre leader, a representative of a South Asian association, a health leader who has been involved in cancer prevention for South Asians, our research team and the acting group, and a script writer. Panel members will meet to discuss what health information to include in the intervention and the strategies required to enhance the cultural sensitivity of the intervention, as well as the linguistic appropriateness and clarity of intervention materials. Panel members will be asked to make recommendations on such strategies.

The development of intervention contents will be guided by the Health Belief Model (HBM), a widely used conceptual framework for developing strategies that induce behavioural changes in chronic disease prevention [30]. For the present intervention, a PowerPoint presentation will first be prepared and presented during the health talks arranged as part of the intervention. The presentation will consist seven sections, namely 1) what is colorectal cancer? 2) risk factors, signs and symptoms, 3) myths and misconceptions, 4) measures for prevention and early detection of CRC, 5) procedures of stool sample collection using a FOBT kit, 6) CRC screening pilot scheme and other service providers in Hong Kong, and 7) implications of a positive test and progression to colonoscopy. After that, a health information booklet will be produced. The PowerPoint presentation will be written in English, while the booklet will first be written in English and later translated into Urdu, Nepali and Punjabi. Advisory panel members and six South Asians (two Indians, two Nepalese and two Pakistanis) as lay persons will be consulted to give feedback on whether the materials present health information in a clear manner, and whether the contents are culturally relevant as well as linguistically appropriate.

A video clip will also be produced based on the storyline and script drafted by the script writer. The aim of the video clip is to highlight the role of younger family members in increasing older South Asian adults’ intention to engage in healthy behaviours. Feedback on clarity and linguistic quality of the script will be sought from the advisory panel members and the six South Asian lay persons. To enhance the cultural sensitivity of the video clip, South Asians will be employed as actors and actresses in the video clip production. Appropriate settings will also be arranged for video shooting, such as the home of a South Asian volunteer and a health clinic. Actors will speak in English during video shooting, after which the video will be dubbed into Hindi with English subtitles.

#### Delivery of intervention

The intervention will involve the assessment of the proportion of dyad participants who undergo FOBT conducted by a family doctor in each group. Prior to the introduction of intervention, our research team will access a list of family doctors enrolled in the CRC screening pilot programme. Family doctors based in the ten chosen districts will be contacted via mail, where the research team will provide an introduction to the aims of the study and request for their consent of collaboration, so that dyad participants can be referred to the consented doctors for FOBT.

Our research team will contact partner organizations in districts allocated as the intervention group for logistical arrangements concerning intervention delivery. During intervention, a health talk will first be held by an intervention instructor hired by the research team. An interpreter will be on hand to provide translations if the instructor can only communicate in English. During the talk, the video clip will be shown to dyad participants, alongside a demonstration on the procedures of stool sample collection for FOBT. Each dyad will then receive a health information booklet at the end of the talk.

For each dyad, a medical appointment for FOBT will be scheduled with a family doctor within 1 month after receiving the intervention. For dyads who express willingness to undergo FOBT, a representative from the partner organizations will be assigned to accompany them in the visit to family doctor for the test and the return stool sample.

#### Wait-list control group

Dyads recruited in districts allocated as the wait-list control group will receive the intervention described above within 2 months after the completion of post-intervention data collection.

### Outcome measures

The outcomes of this study can be classified into five categories based on the RE-AIM framework for the evaluation of the intervention.

#### Reach

The ‘Reach’ of the intervention will be assessed by the recruitment rate, retention rate and the proportion of eligible dyad participants who received and completed the intervention in relation to the targeted sample size of 320 dyads. A record on the number of potential dyad participants approached during subject recruitment, the number of eligible dyads consented to participate in the intervention and the number of dyads who have eventually completed the intervention will be kept for the assessment of the above parameters.

#### Effectiveness

The ‘Effectiveness’ of the intervention will be assessed by three parameters:

- First, the research team will evaluate the proportion of dyads with younger family members (aged between 18 and 49) who are willing to encourage their older family members (aged between 50 and 75) to undergo FOBT through family doctors and ready to assist older adults in stool sample collection for FOBT. The level of willingness and readiness will be assessed using an author-developed questionnaire, where younger family members will rate items based on a five-point rating scale. The ratings obtained from intervention and control dyads will be compared subsequently.
- Second, the research team will also assess the proportion of dyads with older family members (aged between 50 and 75) who have consulted a family doctor for FOBT, completed FOBT and sent their stool samples to the family doctor. A record on the number of older family members of the dyads who have visited family doctor for FOBT will be accessed and the number of stool samples received by the participating family doctors will be recorded. The research team will compare the proportion of participants in the intervention and control dyads who have completed FOBT and provided their stool samples to a family doctor.
- Third, the research team will assess the intervention dyads’ level of satisfaction of the intervention using an author-developed satisfaction questionnaire with a five-point rating scale.

#### Adoption

The ‘Adoption’ of the intervention will be assessed by the proportion of partner organizations agreed to promote and implement our intervention against those approached during the promotion of the intervention. For this purpose, the number of partner organizations approached for the promotion of intervention and that of those agreed to help promote and implement the intervention will be recorded.

Further, the proportion of family doctors enrolled in the CRC screening pilot programme agreed to be engaged in our intervention will also be assessed by recording the number of family doctors approached for their consent to participate in the project against the number of those agreed to be engaged.

#### Implementation

The ‘Implementation’ of the intervention will be assessed by the number of multimedia interventions delivered at partner organizations and the number of visits arranged for older family members of the dyads to consult family doctor. A record on these two parameters will be kept for the assessment.

#### Maintenance

The ‘Maintenance’ of the intervention will be assessed by the level of willingness for persons-in-charge of partner organizations to continue implementing our intervention at their centers after project completion. An author- developed questionnaire with a five-point rating scale will be used for the assessment.

Demographic data, including age, ethnic group, number of people in the household, monthly household income, years of formal education, marital status, employment status, number of years’ residence in Hong Kong, family history of cancer, health insurance status, acculturation and relationship of the dyad members, will also be collected by means of an author-developed questionnaire.

All questionnaires used in outcome measurement will first be developed in English and subsequently translated into Urdu, Nepali and Punjabi by our research staff member of a South Asian origin. Back-translation will be conducted to ensure content and semantic equivalence in the translated questionnaires. A panel consisting of individuals fluent in Urdu, Nepali or Punjabi, and those involved in educating South Asians on health promotion and cancer prevention will be invited to proofread the Urdu, Nepali and Punjabi versions of the questionnaire to ensure the validity of the contents.

### Data collection procedures

Upon receipt of the informed consent, dyad participants will be asked to complete the author-developed questionnaire for the collection of demographic data (T0). At baseline and immediately after the intervention (T1), younger family members of the dyads will be asked to complete the questionnaire that assesses their willingness to encourage older family members to undergo FOBT and their readiness to assist them in collecting stool samples for the test. At T1, dyad participants will also be asked to complete the satisfaction questionnaire to indicate their level of satisfaction of the intervention. A coordinator from our research team will prepare an attendance record, recording the number of older family members of the dyads who attend medical consultation at a family doctor a month post-intervention (T2) and return the stool sample to the family doctor for FOBT 2 months after the medical consultation (T3). Data collection from the dyads in the control group will also be conducted as described above. After project completion, data collectors will approach the persons-in-charge of partner organizations for the completion of a questionnaire enquiring their level of willingness to continue implementing the intervention at their centers.

As a token of appreciation to the dyads’ participation in the intervention, our research team will offer all participants a weighing scale upon completion of baseline data collection.

### Data analysis

Descriptive statistics will be used to report the demographic characteristics of participants in both groups. The baseline characteristics of the two groups will be compared by chi-square or independent t-tests as appropriate. The effectiveness of the intervention will be evaluated by comparing the proportion of younger family members who expressed willingness to encourage their older family members to take up FOBT, as well as their readiness to assist older family members in collecting a stool sample at T1 in both groups. The proportions of older family members who attended medical visit at T2 and reported to have undertaken FOBT at T3 in both groups will also be evaluated. Chi-squared test will be employed for the analysis of these parameters with an intention-to-treat approach. Generalised linear models on the baseline and T1 data will be used to compare the differential changes in two repeated outcome measures for younger family members across the two time points between the two groups, with adjustment for potential confounding variables. The generalised linear modelling will be performed through the Generalised Linear Models Procedure of IBM SPSS statistic v22, depending on the distributions of dependent variables. Descriptive statistics will be used to evaluate dyads’ satisfaction level of the intervention. All statistical tests will be two-sided, with a *p*-value less than 0.05 being considered statistically significant.

### Ethical considerations

Ethical approval has been obtained from the Ethics Committee of the authors’ study institution. Prior to the enrollment of dyad participants, our research team will provide detailed information of the study via an information sheet to explain the aims and detailed procedures of the study. Potential dyad participants will be informed of their rights to withdraw from the study at any stage without penalty. They will be assured of anonymity and confidentiality of any data they provide throughout the study, through the use of participant numbers and the storage of sensitive data in locked cabinets. Dyads will be enrolled into the study only after they have provided their written informed consent to our research staff.

### Research dissemination

Outcomes of this study will be disseminated to healthcare and academic professionals through publications in peer-reviewed journals and presentations at international conferences.

## Discussion

CRC screening is an effective way to detect precancerous lesions in colon and rectum, which enables early CRC detection. This increases the availability of treatment options and likelihood of cancer cure, thereby reducing CRC mortality [31]. Increased utilization of CRC screening would also be likely to reduce the prevalence of CRC worldwide, and should therefore be encouraged. Nevertheless, as indicated above, South Asian ethnic minorities were found to exhibit a low uptake of cancer screening, primarily due to their inadequate knowledge on cancer prevention and cultural beliefs. Moreover, our previous study also revealed that only 10% of South Asians aged 50 or above had previously undergone CRC screening [14], implying that the older generation of South Asian ethnic minorities tend not to utilize CRC screening services available to them. As CRC risks were shown to increase with age [4], the development and implementation of interventions, primarily targeting older South Asian adults, effective in promoting CRC screening uptake would be of paramount importance. Since older South Asian older were found to often rely on their younger family members in the acquisition of health-related information [27], a family-based approach would be required in the development of intervention that aims to raise the awareness on the importance of CRC screening in CRC prevention among older South Asian older adults. Here, a CRC screening intervention that targets both the younger and older members of families is proposed to enhance its effectiveness in disseminating information on CRC prevention among older South Asian older and encouraging the utilization of CRC screening. To the best of our knowledge, this is the first study dedicated to assessing the effectiveness of a family-based intervention to increase CRC screening uptake among South Asian ethnic minorities within a Chinese community.

The potential impact of study findings is two-fold. First, they would provide further evidence of the benefits of utilizing multimedia and family-based approaches in intervention development to enhance the effectiveness of health promotion interventions. Indeed, multimedia interventions were previously shown effective in enhancing the knowledge of ethnic minorities on cancer and improving their self-efficacy in undergoing cancer screening [32, 33]. Likewise, a systematic review also demonstrated the benefits of family-based interventions on improving the outcomes of diabetic patients [26]. Nevertheless, there is a lack of data on whether interventions that combined family-based and multimedia elements would be effective in knowledge dissemination on CRC and its prevention among South Asian ethnic minorities, thereby subsequently enhancing CRC screening uptake. This study could potentially address this knowledge gap, and serve as a basis for the development of future interventions that aim to promote effective prevention of other chronic diseases, including obesity, cardiovascular diseases and diabetes, among South Asian ethnic minorities.

Second, the study would potentially provide evidence of the applicability and acceptability of a family-based multimedia intervention conducted among South Asian ethnic minorities in a community setting. Effectiveness of this intervention in enhancing CRC screening uptake among older South Asian adults could provide reference for potential incorporation of the intervention in the existing support services provided by various organizations supporting local South Asians, thereby enhancing the healthcare and support services available to these individuals. This could help address the current ethnic disparities in CRC screening uptake.

Despite the potential impacts of this study, it suffers one major limitation that requires acknowledgement. The study will recruit only Indians, Pakistani and Nepali, the three largest South Asian populations in Hong Kong, as subjects. Other smaller South Asian populations in Hong Kong, such as Bangladeshis and Sri Lankans, will be excluded due to our limited knowledge on South Asian languages, which is restricted only to Urdu, Nepali and Punjabi, for the preparation of intervention materials. Such exclusion would limit the ‘Reach’ of the intervention. Nevertheless, with the scarcity of data on the effectiveness of family-based and multimedia interventions on increasing the intention of South Asian ethnic minorities to take up CRC screening, this study would provide useful data to fill this research gap, serving as a case study for the implementation of such interventions targeting South Asian ethnic minorities within the community.

## Summary

Increased utilization of CRC screening, especially among older adults, is vital to combating the increasing prevalence of CRC worldwide. With South Asian ethnic minorities previously shown to exhibit a low screening uptake, it is of great importance to implement interventions to enhance their knowledge on CRC and its preventive measures. The proposed intervention, using a combination of family-based and multimedia approaches, would potentially enable older South Asian adults to acquire a better understanding on the preventive measures of CRC and show greater self- efficacy in undergoing CRC screening. Findings of this study would shed light on the applicability of incorporating this intervention in the existing support services available to local South Asian ethnic minorities, potentially allowing them to prevent CRC more effectively.

## Trial status

The trial was scheduled to start on 1st September 2018, and is expected to be completed by 31st August 2020.

## Additional file

Additional file 1: The SPIRIT checklist. (DOC 121 kb)

## Abbreviations

CRC: Colorectal cancer
FOBT: Fecal occult blood test
HBM: Health belief model
RE-AIM: Reach-Effectiveness-Adoption-Implementation-Maintenance
