# Correction to: A family-based multimedia intervention to enhance the uptake of colorectal cancer screening among older south Asian adults in Hong Kong: a study protocol for a cluster randomized controlled trial

**DOI:** 10.1186/s12889-021-12243-0

**Published:** 2021-12-03

**Authors:** Winnie K. W. So, Bernard M. H. Law, Kai Chow Choi, Dorothy N. S. Chan, Carmen W. H. Chan

**Affiliations:** The Nethersole School of Nursing, The Chinese University of Hong Kong, Shatin, Hong Kong, China

**Correction to: BMC Public Health 19, 652 (2019)**

**https://doi.org/10.1186/s12889-019-6995-7**

It was highlighted that in the original article [1], in the Methods sections of the Abstract and the Article, it was erroneously reported that the number of districts in Hong Kong that will be involved in cluster randomisation in the proposed randomised controlled trial is ten instead of six. This correction article shows the corrections below.

**Section: Methods (Abstract).**

Three hundred and twenty South Asian dyads, comprising an older adult aged between 50 and 75 and a younger family member aged between 18 and 49, will be recruited in six districts in Hong Kong through community organizations that provide support services for South Asians in local communities.

**Section: Methods – Study settings.**

Interventions will be conducted in various South Asian community centres, ethnic minority associations and non-governmental organizations that provide support services for South Asians within local communities in six districts in Hong Kong.

**Section: Methods – Randomization.**

A cluster randomization approach will be utilized in this study. In previous community-based projects, we had identified six districts in Hong Kong populated with a large number of South Asians. These districts will be randomized into either the intervention or wait-list control group, with three districts allocated to each group respectively.

**Section: Methods – Delivery of intervention.**

Family doctors based in the six chosen districts will be contacted via mail, where the research team will provide an introduction to the aims of the study and request for their consent of collaboration, so that dyad participants can be referred to the consented doctors for FOBT.
